# Toward the Rational Design of Ion‐Conducting Ionic Liquid‐Incorporated Metal–Organic Framework Hybrids

**DOI:** 10.1002/asia.202500966

**Published:** 2025-12-17

**Authors:** Yukihiro Yoshida, Tuo Di, Hiroshi Kitagawa

**Affiliations:** ^1^ Division of Chemistry Graduate School of Science Kyoto University Kyoto Japan

**Keywords:** ionic liquid, ionic conduction, lithium‐ion conduction, metal–organic framework

## Abstract

Solid‐state ionics have been the subject of intense research because of their possible applications as solid electrolytes for all‐solid‐state electrochemical devices. Ionic liquid‐introduced metal–organic frameworks (IL@MOFs) are an emerging class of hybrid solids with superior ionic conductivity, in which the migration of component ions of ILs in the pores is responsible for ionic conduction. In this review, we present an overview of the development of ion‐conducting IL@MOF hybrids from the perspective of synthetic methodologies to incorporate ILs into MOFs and the ion‐conducting behavior controlled by the IL filling level, ion species of ILs, and MOF structures such as pore size and pore surface state. Finally, we devote attention to the Li^+^‐ion conduction, especially the Li^+^‐ion transport number, of the pore‐encapsulated Li^+^‐containing ILs directed toward applications in all‐solid‐state lithium‐ion batteries.

## Graphical abstract

We present the overview of the development of ionic liquid‐introduced metal–organic framework (IL@MOF) hybrids from the perspectives of synthetic methodologies to incorporate ILs into MOFs and ionic conduction controlled by IL filling level, ion species of ILs, and MOF structures such as pore size and pore surface state. Additionally, the Li^+^‐ion conduction, especially the Li^+^‐ion transport number, of pore‐encapsulated Li^+^‐containing ILs is presented toward applications in all‐solid‐state lithium‐ion batteries.

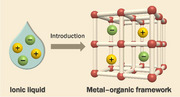

## 1 Introduction

**FIGURE 1:**
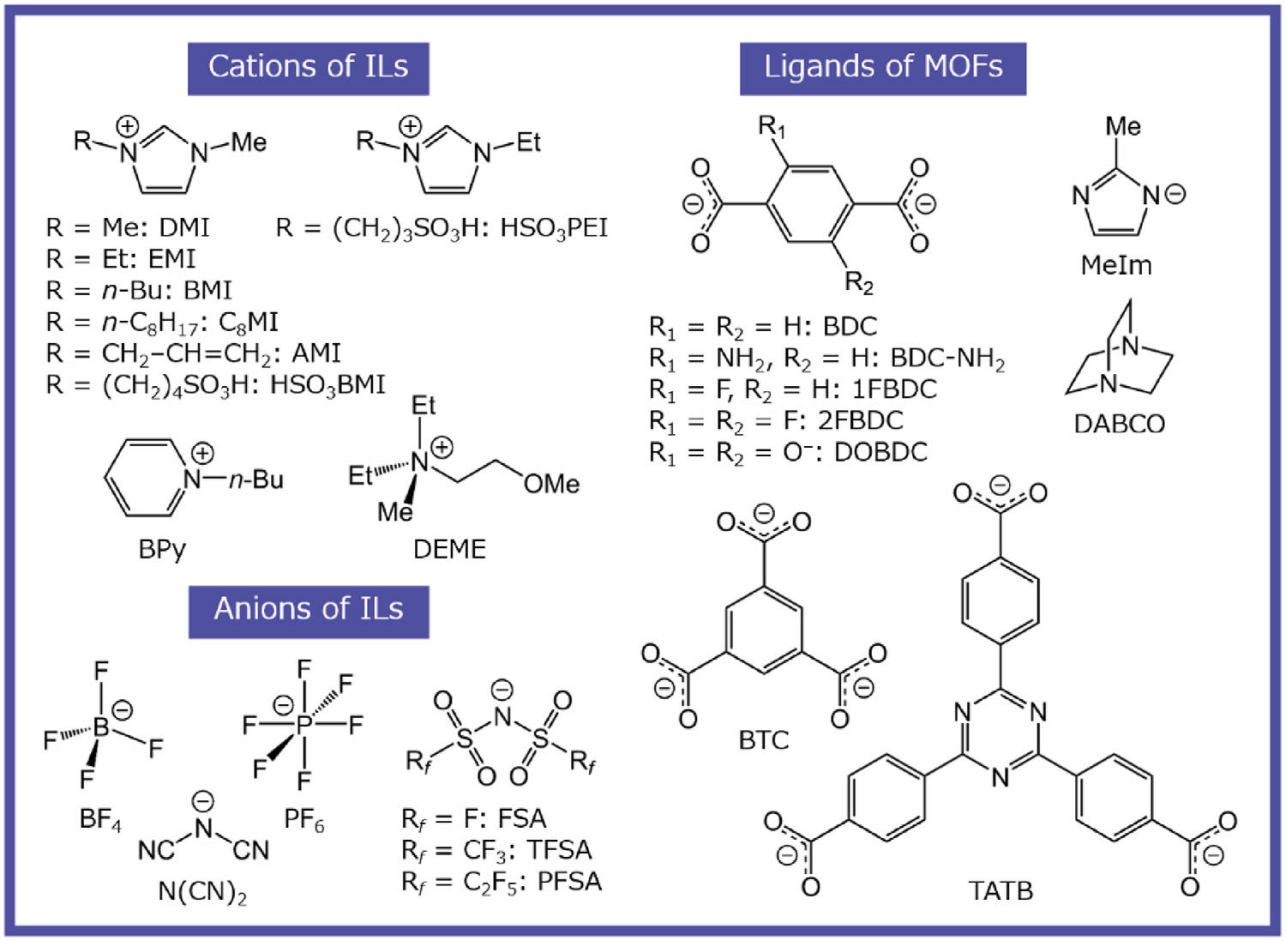
Molecular structures of typical component ions of ionic liquids (ILs) and ligands of metal–organic frameworks (MOFs) presented in this review.

The history of ionic liquids (ILs) dates back to the 19th century, when Gabriel and Weiner reported that ethanol ammonium nitrate melts at 52–55°C as early as 1888 [1]. This is the first salt that satisfies the most consensually accepted definition of IL, namely “a molten salt composed entirely of ions with a melting temperature below 100°C.” Whereas pioneering works by Walden [2], whose name is preserved in the Walden inversion, opened the door to IL research, the widespread use of ILs in synthetic, physical, electrochemical, and catalytic chemistry over the past 30 years originates in the first water‐ and air‐stable room‐temperature (RT) imidazolium‐based ILs with BF_4_ and AcO anions, reported by Wilkes and Zaworotko in 1992 [3]. ILs containing the bis(trifluoromethanesulfonyl)amide (TFSA) anion (Figure 1), which is currently the most commonly used anion in electrochemistry, were first reported by Grätzel and his workers in 1996 [4]. TFSA‐based ILs not only have moderately high ionic conductivity and low viscosity but also exceptional hydrophobicity, which allows them to be handled under ambient conditions. Owing to their excellent thermal and electrochemical durabilities, TFSA‐based ILs have been extensively studied as electrolytes for capacitors, batteries, and field‐effect transistors [5, 6, 7, 8].

It is clear that the electrostatic interactions between the ions in ILs with high ion concentrations are responsible for their negligible vapor pressure. Although this liquid property indicates the serious drawback of the difficulty in the purification of ILs by distillation, it has the advantage of being non‐flammable and eliminating liquid loss due to volatilization. However, it is noteworthy that the problem of leakage, which is an important issue for the packaging and portability of devices, still remains because ILs are fluid like water and organic solvents. To address these drawbacks, two approaches have been pursued over the past two decades in the field of polymer science: solid polymers embedded with ILs [9, 10] and polymeric ILs [11, 12]. The latter, which consists of polymerized cations and/or anions and is generally in the solid state despite its name, exhibited reduced ionic conductivity due to the presence of immobile ions. Additionally, the serious obstacle lies in the difficulty of the chemical inclusion of polymerization sites into the precursor ions.

Metal–organic frameworks (hereafter referred to as MOFs; honored with the 2025 Nobel Prize in Chemistry) are crystalline porous materials with excellent designability that surpasses those of conventional porous materials such as activated carbon, porous silica, and zeolites; namely, their framework structure varies largely depending on the selection of their constituent metal ions (or nodes) and multidentate ligands [13, 14, 15]. As an example, a variety of framework structures can be constructed by terephthalate (BDC^2–^) anions depending on the coordinated metal ions (i.e., MIL‐53(Al), MIL‐101(Cr), MOF‐5(Zn), UiO‐66(Zr), and so on) [16]. The lattice constant (i.e., pore size) of isoreticular Zr‐based MOFs can be continuously tuned by changing the mixing ratio of the bidentate dicarboxylate anions with different lengths as linkers [17, 18], whereas the framework structures of some MOFs vary markedly depending on the guest species introduced into the pores [19]. Thus, it is conceivable that judiciously designed combinations of ILs as guests and MOFs as hosts will contribute to the development of solid‐state ionics with superior or novel properties compared with existing solids.

**FIGURE 2:**
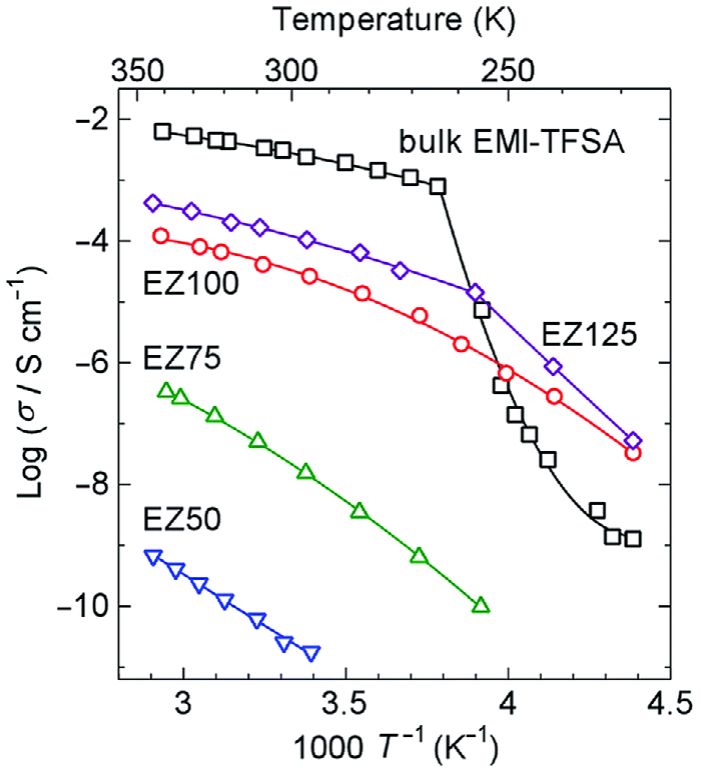
Arrhenius plots of the ionic conductivity (*σ*) of (EMI)(TFSA)@ZIF‐8 hybrids with volumetric occupancy *f* = 0.5 (EZ50), *f* = 0.75 (EZ75), *f* = 1.0 (EZ100), and *f* = 1.25 (EZ125), together with that of bulk (EMI)(TFSA) (bulk EMI‐TFSA). The solid lines are drawn as guides for the eye. Adapted with permission from Ref. [20]. Copyright 2015 Royal Society of Chemistry.

Since Fujie and Kitagawa et al. reported that (EMI)(TFSA)‐incorporated microporous MOF, [Zn(MeIm)_2_] (ZIF‐8), exhibited a moderately high ionic conductivity (*σ*_RT_ = 2.6 × 10^−5^ S cm^−1^; Figure 2) in 2015 [20], considerable research has been conducted worldwide to explore IL@MOF hybrids (note: the “@” symbol indicates the state in which the IL is enclosed within the pore without any chemical bonds), primarily aiming at the potential application as solid electrolytes. Notably, ILs introduced into MOF pores do not solidify down to lower temperatures because of the increased surface energy caused by the nanosize effect [21]. In fact, several studies have demonstrated that the ionic conductivity of IL@MOFs, which is inferior to that of the corresponding bulk IL at RT, is higher than that of the bulk IL at low temperatures owing to the retention of the liquid state [20, 22]. It is also known that the small Li^+^ ion (ionic radius: 0.76 Å) contained in IL introduced into MOF pores preferentially transports through the nanospaces, especially apertures connecting neighboring pores, compared to the bulk state, resulting in the high Li^+^‐ion transport number ($t_{Li^{+}}$), as shown below [23, 24, 25, 26, 27, 28, 29].

## 2 Methods of Introducing ILs Into MOFs

**FIGURE 3:**
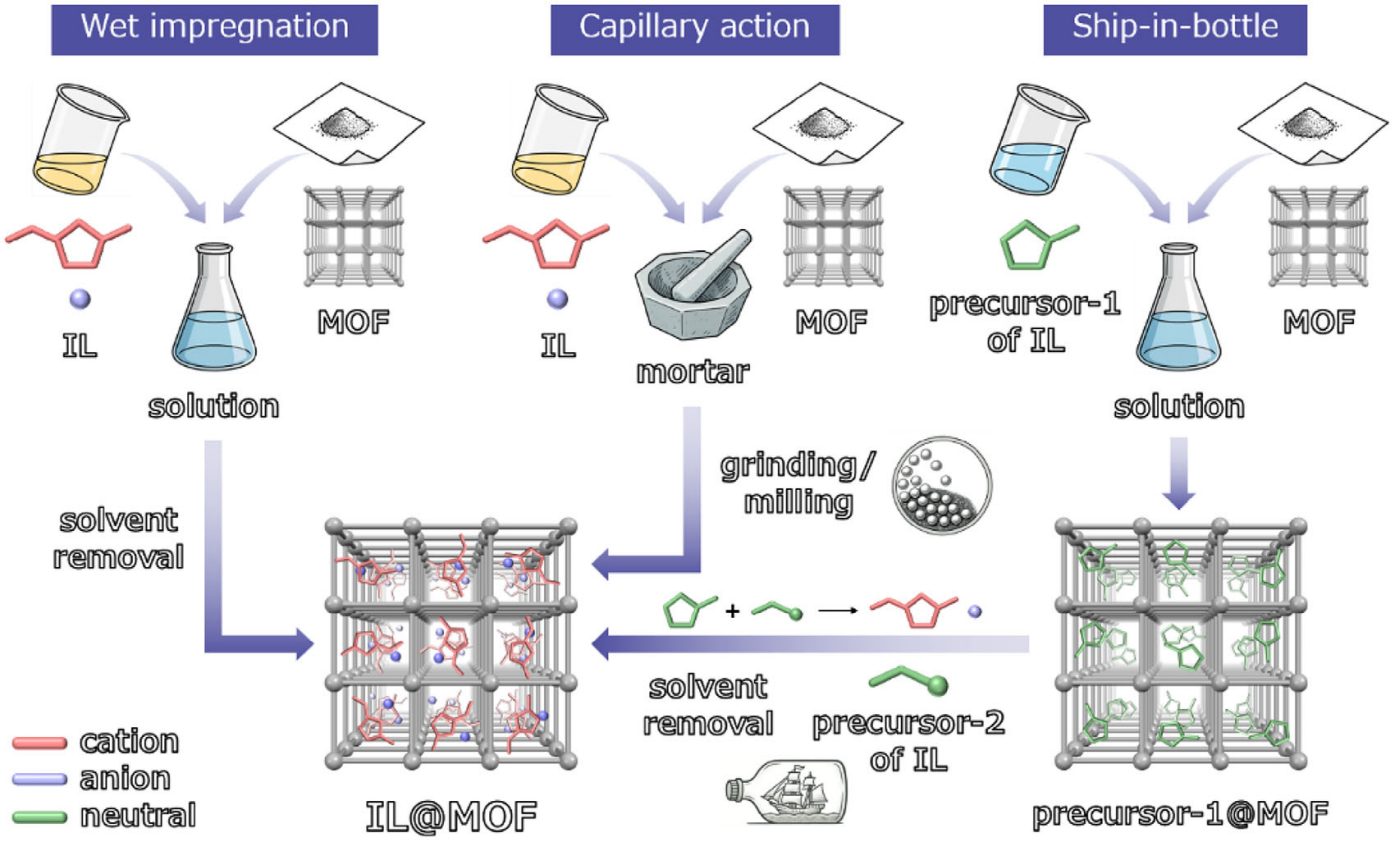
Representation of different post‐synthesis methods used in the preparation of IL@MOF hybrids, namely, wet impregnation, capillary action (or dry milling), and ship‐in‐bottle (or tandem) methods.

In general, ILs can be introduced into MOF pores either by the ionothermal method, in which the structure of MOFs is constructed in the IL while confining the IL in the pores, or by the post‐synthesis method, in which the IL is introduced into pre‐built MOFs that were degassed by thermal activation under vacuum [30, 31, 32, 33]. It is noteworthy that, in the former method, IL may act merely as a reaction solvent or provide only one of the component ions as a linker or a guest ion in the MOFs [34]. Thus, the latter method is the prevailing approach for synthesizing IL@MOF hybrids at the present stage. The post‐synthesis approach includes (1) the wet impregnation [35] and (2) the capillary action (also referred to as the dry milling) [36], as shown in Figure 3. In addition, a method in which neutral precursors of ILs (e.g., heterocyclic bases and alkyl halides) are introduced into MOF pores followed by alkylation (or Menschutkin) reaction, called the tandem method [37] or the ship‐in‐bottle method [38], has also been proposed (Figure 3). Method (1) involves impregnation of the MOF in a polar solvent, such as water, alcohols, or acetonitrile, in which the IL is dissolved. Although the use of a large excess of IL ensures reproducibility of the amount introduced, there is a risk that the reaction solvent will also be introduced into the MOF pores. On the other hand, in method (2), the introduction of IL into the MOF pores is mechanically achieved by mortar grinding or ball milling, which prevents contamination of the reaction solvent; however, there is a concern that the IL is not homogeneously filled into the pore space of the MOF. The influence of the introduction method on the filling level of ILs has been reported for [Cr_3_O(F, OH)(H_2_O)_2_(BDC)_3_] (MIL‐101(Cr)); the ship‐in‐bottle method in water gives a higher volumetric filling level (*f* = 0.95) than that by the wet impregnation method in ethanol (*f* = 0.71), resulting in the higher ionic conductivity by more than four orders of magnitude [39]. In many reported papers, the amount of introduced IL (i.e., filling level) has been estimated from the nominal ratio, weight change, elemental analysis (e.g., combustion and energy‐dispersive X‐ray spectroscopy), and gas sorption measurements.

**FIGURE 4:**
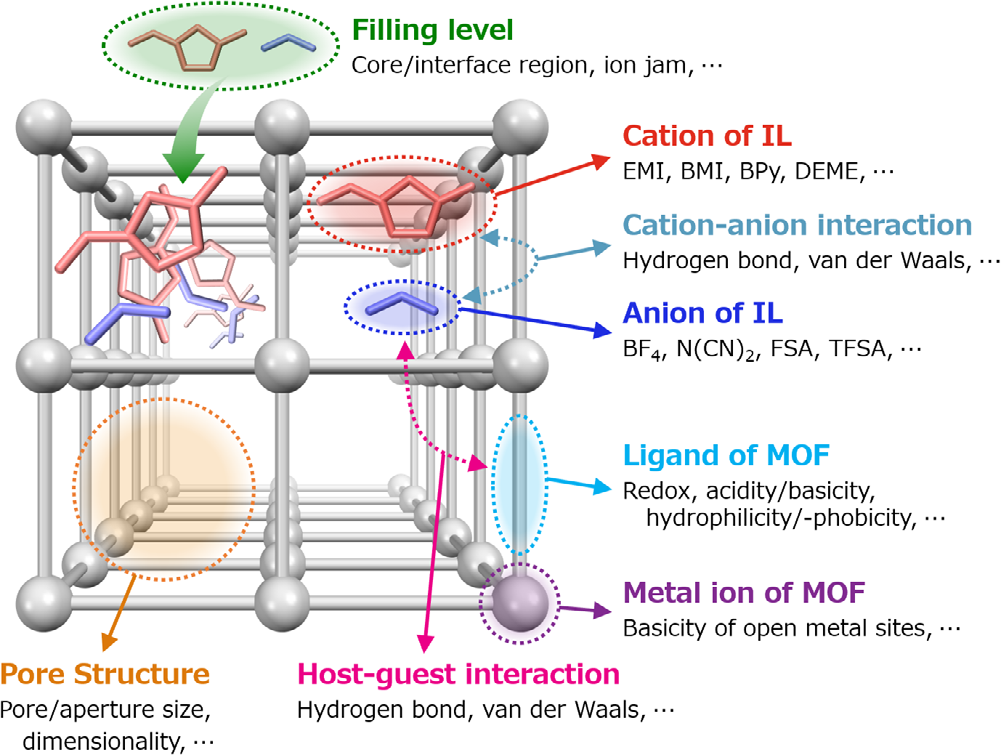
Classification of the factors influencing the ion‐conducting behavior of IL@MOF hybrids. Note that these factors are highly correlated with each other.

In the following, we present the current status of accumulated knowledge regarding the factors that affect the ion‐conducting behavior of IL@MOF hybrids, along with the underlying principles and strategies for controlling the transport properties as illustrated in Figure 4.

## 3 Effect of Filling Level of ILs

**FIGURE 5:**
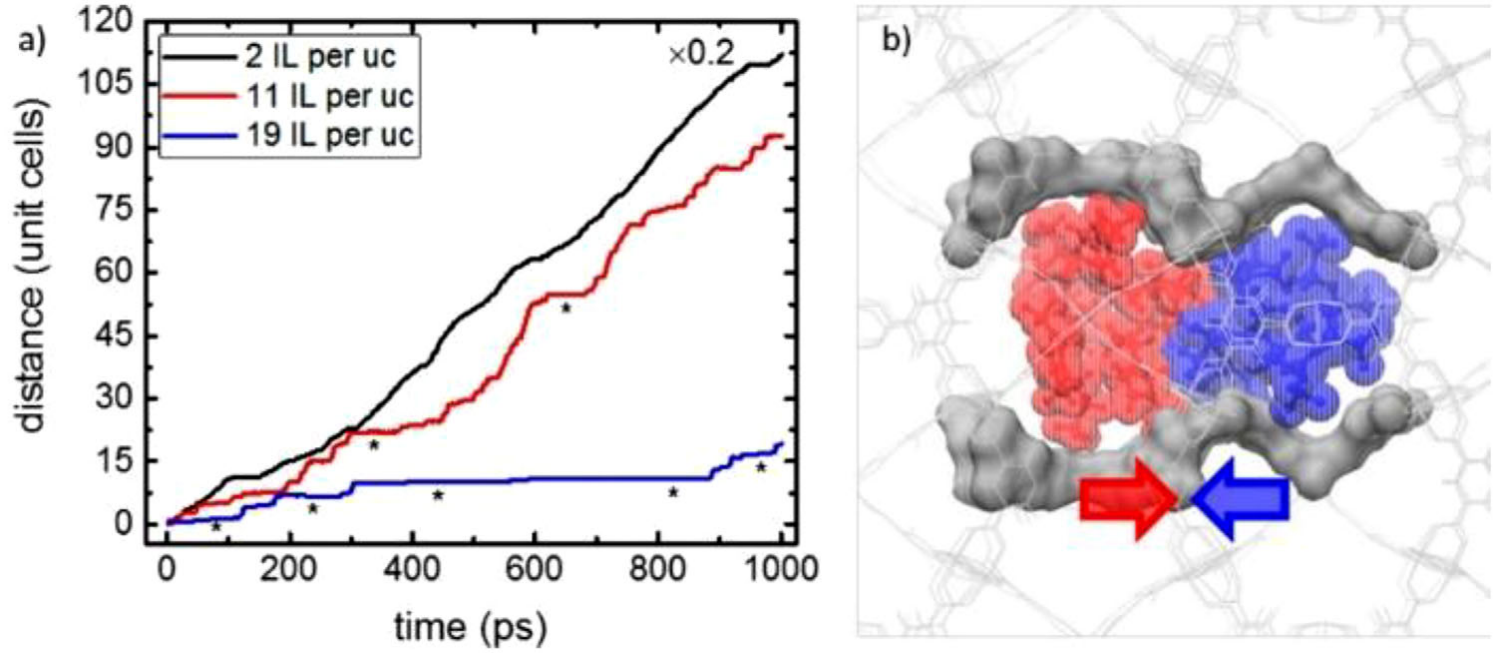
Trajectories of BMI cations of (BMI)(TFSA) in the pores of HKUST‐1 (cubic lattice with a lattice constant of 2.6 nm) with a concentration of 2 (black), 11 (red), and 19 (blue) ion pairs per unit cell. Asterisks indicate the transient immobilization near the apertures connecting neighboring pores. (b) Sketch of a blocked MOF pore (gray), where BMI cations (red) and TFSA anions (blue) endeavor to drift to the right and left, respectively, under a horizontally applied electric field of 7.5 V nm^−1^. Adapted with permission from Ref. [40]. Copyright 2019 American Chemical Society.

As a general trend, the ionic conductivity of IL@MOF hybrids increases with increasing IL filling level, as a consequence of the increased ion concentration in the pores. However, several studies have demonstrated that ionic conductivity exhibits a distinct decrease as the filling factor approaches *f* = 1 [22, 40, 41, 42, 43]. Molecular dynamics (MD) simulations could reproduce the time course (trajectory) of the IL configuration in the MOF pores. Heinke et al. reported that the component ions of (BMI)(TFSA) introduced in the pores of [Cu_3_(BTC)_2_] (HKUST‐1) migrate in proportion to time at the low filling level (two ion pairs in a unit cell; “2 IL per uc” in Figure 5a), whereas the increase in the filling level causes the ion jam [44] near the apertures connecting the pores (19 ion pairs in a unit cell; “19 IL per uc” in Figure 5a,b) [40]. This can be attributed to the bulkiness of the component ions (BMI: 195 Å^3^, TFSA: 196 Å^3^) compared to the aperture diameter (ca. 0.9 nm), despite their orientational degrees of freedom.

**FIGURE 6:**
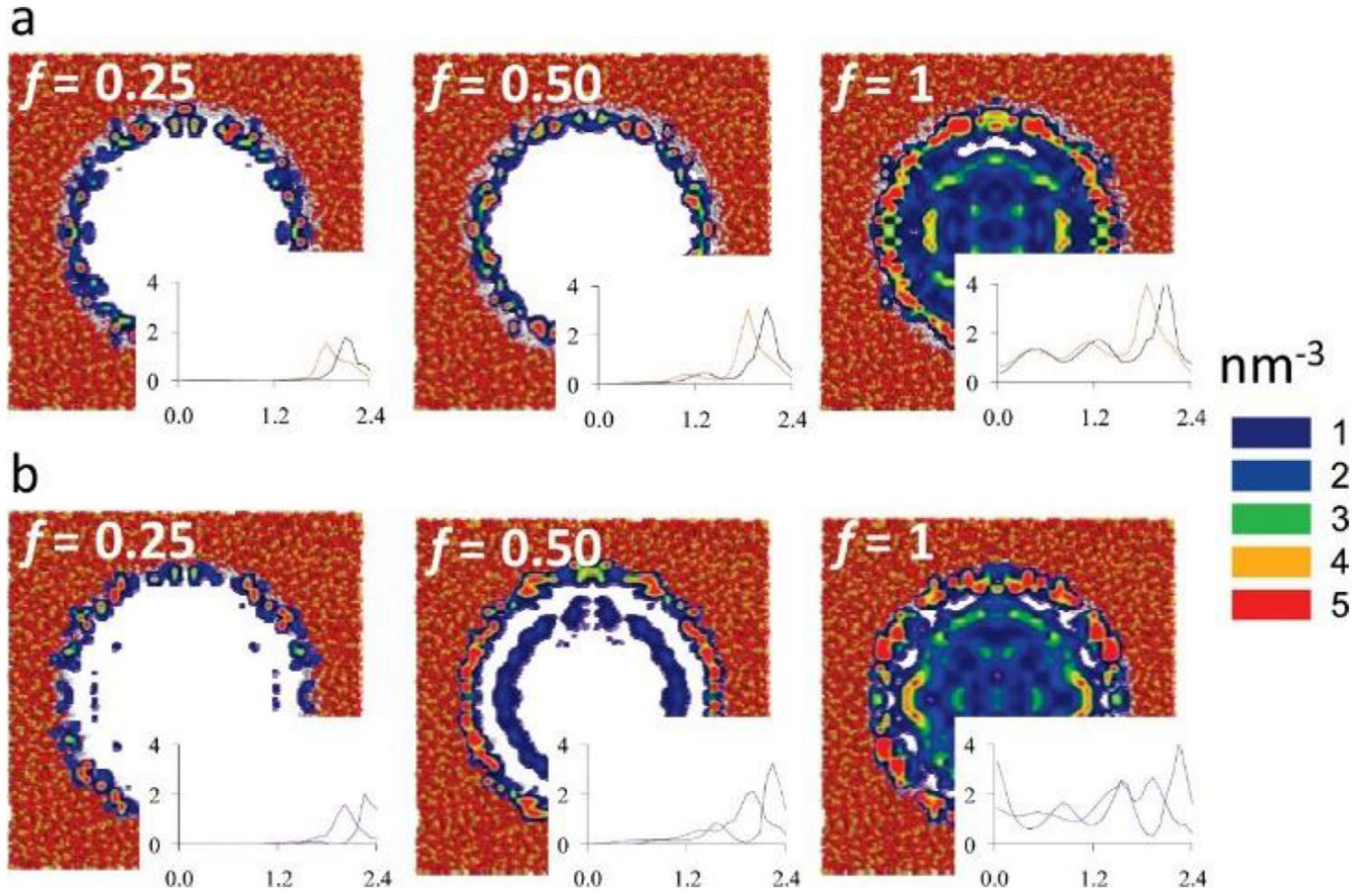
Contour plots showing the density distribution of (a) TFSA anions and (b) BMI cations of (BMI)(TFSA) in a nanoporous hydroxylated silica with a diameter of 4.8 nm, predicted by MD simulations. The filling factors are *f* = 0.25 (left), *f* = 0.5 (middle), and *f* = 1.0 (right). Orange and red segments denote silicon and oxygen atoms, respectively, whereas the white segments are hydrogen atoms that delimit the pore surface. For each anion contour plot, the insets show the radial density profiles in nm^−3^ for the fluorine (orange line) and oxygen (black line) atoms of the anion. For each cation contour plot, the insets show the radial density profiles in nm^−3^ for the nitrogen atom (blue line) and the terminal carbon atom of the *n*‐butyl chain (purple line) of the cation. Adapted with permission from Ref. [45]. Copyright 2011 American Chemical Society.

Furthermore, MD simulations predicted that (BMI)(TFSA) preferentially occupies the interface region near the pore surface of mesoporous silica (Figure 6) [45, 46]. As the filling factor increases further, the component ions begin to occupy the core region and are little affected by the potential of the pore surfaces located farther than 1 nm. In other words, ILs occupying the core region in mesoporous MOFs with diameters larger than 2 nm behave similarly to the corresponding bulk state.

## 4 IL Species Dependence

To date, the ILs used to prepare IL@MOF hybrids are limited primarily to those composed of 1‐alkyl‐3‐methylimidazolium (RMI) as cations and TFSA and halides (i.e., Cl and Br) as anions. Thus, the effect of IL species introduced into the MOF pores on the ionic conductivity of IL@MOF hybrids has been largely unexplored.

### 4.1 Cation Species Dependence

The dependence of cation species (EMI, BMI, and BPy) on ionic conductivity was investigated for Na^+^‐containing ILs introduced into the pores of MIL‐101(Cr) with BDC ligands bearing an SO_3_Na group [47]. Although the transport number of each ion is unclear, the ionic conductivity at 150°C increases when the BMI cation is replaced by the EMI cation (1.8 × 10^−3^ S cm^−1^ to 8.7 × 10^−3^ S cm^−1^ for the TFSA anion). Replacing the BPy cation with the EMI cation leads to an increase in ionic conductivity by approximately one order of magnitude (1.3 × 10^−3^ S cm^−1^ to 1.3 × 10^−2^ S cm^−1^ for the BF_4_ anion). This cation dependence is similar to that of the bulk liquid case [48, 49, 50]. We note that Li^+^‐containing ILs introduced into the pores of UiO‐66(Zr) with BDC ligands bearing an SO_3_Li group show a RT ionic conductivity of approximately 10^−4^ S cm^−1^ and an activation energy (ca. 0.3 eV) regardless of the cation species (EMI or DEME) [29]. Given that the ionic conductivity of EMI‐based ILs is more than three times higher than that of the corresponding DEME‐based ILs in the bulk state [51, 52], this result is indicative of the significant contribution of Li^+^ ions present in both the IL and MOF to ionic conduction ($t_{Li^{+}}$ ≈ 0.3). In addition, the fact that the quaternary ammonium cation, DEME (239 Å^3^), is more flexible than the heterocyclic cation, EMI (149 Å^3^), could facilitate the passage of DEME cations through the apertures (ca. 0.5 nm) connecting neighboring pores.

**FIGURE 7:**
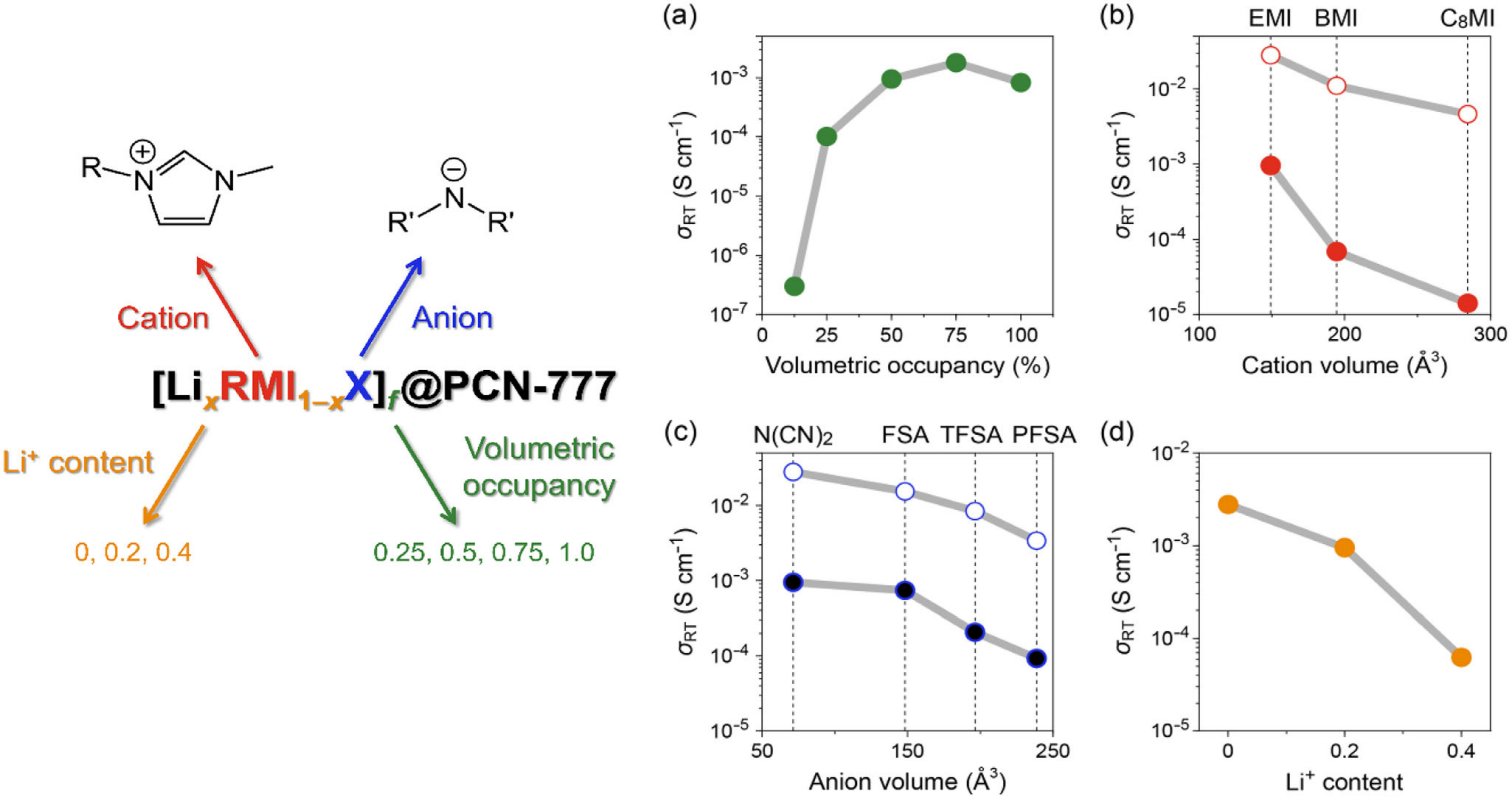
RT ionic conductivity (*σ*_RT_) of Li*_x_* RMI_1–_ *_x_* X@PCN‐777(Zr) hybrids as a function of (a) volumetric occupancy (*f*) of Li_0.2_RMI_0.8_[N(CN)_2_], (b) alkyl chain length of cations (*f* = 0.5, *x* = 0.2, X = N(CN)_2_; ●: Li_0.2_RMI_0.8_[N(CN)_2_]@PCN‐777(Zr) hybrids, ○: bulk Li_0.2_RMI_0.8_[N(CN)_2_]), (c) perfluoroalkyl chain length of anions (*f* = 0.5, *x* = 0.2, R = Et; ●: Li_0.2_EMI_0.8_X@PCN‐777(Zr) hybrids, ○: bulk Li_0.2_EMI_0.8_X), and (d) Li^+^ content (*x*) (*f* = 0.5, R = Et, X = N(CN)_2_) [53].

Our group introduced Li^+^‐containing IL, prepared by dissolving 20 mol% of Li[N(CN)_2_] in ILs composed of RMI cations with different alkyl chain lengths and N(CN)_2_ anions, into the mesoporous [Zr_6_O_4_(OH)_10_(H_2_O)_6_(TATB)_2_] (PCN‐777(Zr); pore size: 3.5 nm) by dry milling (Figure 7) [53]. As shown in Figure 7b, the RT ionic conductivity of Li_0.2_RMI_0.8_[N(CN)_2_]@PCN‐777(Zr) hybrids decreases with elongating the alkyl chain (EMI: 9.5 × 10^−4^ S cm^−1^ to C_8_MI: 1.4 × 10^−5^ S cm^−1^), possibly caused by the increased van der Waals interactions between alkyl chains and the decreased ion concentration due to the alkyl chain elongation as in the case of bulk ILs [48, 50].

### 4.2 Anion Species Dependence

As mentioned above, the component anions of ILs used in IL@MOF hybrids have been primarily limited to TFSA and halides (Cl and Br); therefore, studies on the anion species dependence of ionic conductivity remain less explored than those on the cation species.

The variation in the ionic conductivity of anion species (TFSA, BF_4_, and PF_6_) was investigated in Na^+^‐containing ILs introduced into MIL‐101(Cr) with BDC ligands bearing an SO_3_Na group [47]. The ionic conductivity at 150°C decreases when the TFSA anion is replaced with the PF_6_ anion (1.8 × 10^−3^ S cm^−1^ to 1.1 × 10^−4^ S cm^−1^ for the BMI cation), whereas the replacement of the TFSA anion with the BF_4_ anion leads to the increase in ionic conductivity (8.7 × 10^−3^ S cm^−1^ to 1.3 × 10^−2^ S cm^−1^ for the EMI cation). We have systematically investigated the effect of the perfluoroalkyl chain length of the amide anions on the ionic conductivity of the IL@PCN‐777(Zr) hybrids (Figure 7c) [53]. Similar to the bulk ILs [52, 54], the RT ionic conductivity of Li_0.2_EMI_0.8_[N(C*_n_* F_2_ *_n_*_+1_SO_2_)_2_]@PCN‐777(Zr) hybrids (*n* = 0, 1, and 2) decreases with elongating the perfluoroalkyl chain (FSA (*n* = 0): 7.4 × 10^−4^ S cm^−1^ to PFSA (*n* = 2): 9.3 × 10^−5^ S cm^−1^). Both values are lower than that of the Li_0.2_EMI_0.8_[N(CN)_2_]@PCN‐777(Zr) hybrid formed with N(CN)_2_ anions (9.5 × 10^−4^ S cm^−1^).

Whereas most of the conductivity variation in IL@MOFs hybrids is quite similar to that of the bulk ILs [52, 54], it has been reported that the RT ionic conductivity of (EMI)(SCN) introduced into the pores of MIL‐101(Cr) (1.2 × 10^−3^ S cm^−1^ at *f* = 1.0) is higher than that of (EMI)[N(CN)_2_]@MIL‐101(Cr) (4.1 × 10^−4^ S cm^−1^ at *f* = 1.0) [55]. Although the aperture size of MIL‐101(Cr) (< ca. 1.5 nm) is large enough for both linear SCN (50 Å^3^) and bent N(CN)_2_ (71 Å^3^) anions to pass through, the difference in ion shape may affect the ion jam [44] near the aperture.

Relative to the TFSA anion (196 Å^3^), which is similar in size to the BMI cation (195 Å^3^), the transport number of the TFSA anion is comparable to that of the BMI cation in the bulk (BMI)(TFSA) [54]. However, MD simulations showed that the transport number of the TFSA anion of (BMI)(TFSA) introduced into the pores of UiO‐66(Zr) with a narrow aperture (ca. 0.53 nm) falls to less than one‐quarter of that of the bulk IL, possibly because of the rigid nature of the TFSA anion compared to the BMI cation with a flexible *n*‐butyl group [43].

## 5 MOF Species Dependence

It is plausible that the porous properties (i.e., size and surface conditions) of MOFs have a significant effect on the transport behavior of ILs introduced into the pores. Although techniques and knowledge have been steadily accumulated to systematically fabricate isoreticular MOFs with different metal species, ligand sizes, and functional groups attached to the ligands, studies on their application to control and improve the ionic conductivity of IL@MOF hybrids remain scarce.

### 5.1 Pore Size Dependence

When the dicarboxylate bridging ligands that constitute the Zr‐based MOFs are elongated from benzene (UiO‐66(Zr)) to biphenyl (UiO‐67(Zr)), and even to 2’,5’‐dimethyl‐*p*‐terphenyl (PCN‐56(Zr)), the ionic conductivity at 90°C of (Et_4_N)(TFSA) (melting point in bulk state: 102°C; this salt is not regarded as IL according to the most consensually accepted definition of IL, “melting point below 100°C”) shows a slight increase (5.9 × 10^−5^ S cm^−1^ to 7.7 × 10^−5^ S cm^−1^) [56]. It is possible that the increase in pore size due to ligand elongation is one of the main reasons for the increase in ionic conductivity. The increase in RT ionic conductivity of (BMI)(TFSA) in the pores has also been observed by elongating the dicarboxylate bridging ligands from benzene (UiO‐66(Zr)) to naphthalene (DUT‐52 (Zr)), and even to biphenyl (UiO‐67(Zr)) (ca. 4 × 10^−8^ S cm^−1^ to ca. 9 × 10^−8^ S cm^−1^) [43]. MD simulations predicted that the increase in pore and aperture sizes promoted ion diffusion of both component ions of (BMI)(TFSA) as a consequence of the relief of the ion jam [44].

**FIGURE 8:**
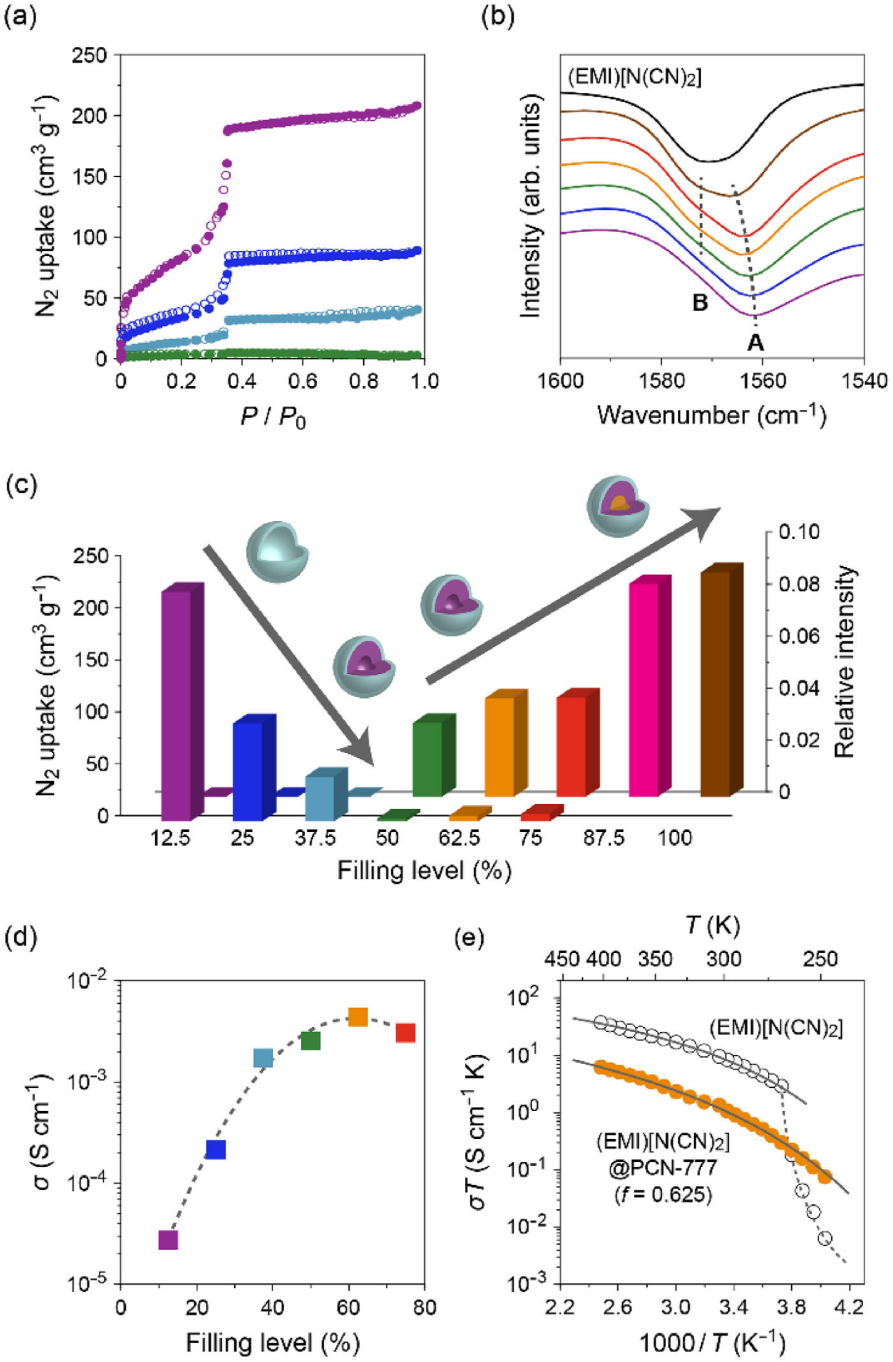
(a) Nitrogen gas adsorption (closed circles) and desorption (open circles) isotherms of (EMI)[N(CN)_2_]@PCN‐777(Zr) hybrids measured at 77 K. (b) FT‐IR spectra of hybrids and bulk (EMI)[N(CN)_2_] in the range of the C–N rocking vibrational mode. The right (**A**) and left (**B**) dotted lines show Bands **A** and **B**, respectively (see text for details). (c) Maximum nitrogen uptake (front line; Figure 8a) and relative intensity of Band **B** to Band **A** (rear line; Figure 8b) of hybrids as a function of volumetric occupancy (*f*). (d) RT ionic conductivity (*σ*_RT_) of hybrids as a function of *f*. (e) Temperature dependence of *σ*_RT_ *T* of (EMI)[N(CN)_2_]@PCN‐777(Zr) with *f* = 0.625 (●) and bulk (EMI)[N(CN)_2_] (○). Purple: *f* = 0.125, blue: *f* = 0.25, pale blue: *f* = 0.375, green: *f* = 0.5, orange: *f* = 0.625, red: *f* = 0.75, pink: *f* = 0.875, brown: *f* = 1.0. Adapted with permission from Ref. [22]. Copyright 2019 Wiley‐VCH.

As mentioned above, it is expected that the ions occupying the core region more than 1 nm from the pore surface will not be significantly affected by the pore surface potential [45, 46]. In other words, ILs introduced into MOFs with pore diameters larger than 2 nm can exhibit ion diffusion comparable to that in the bulk state, despite the IL@MOF hybrids maintaining their solid form. Our group prepared a series of IL@MOF hybrids composed of mesoporous PCN‐777(Zr) and the highly ion‐conductive (EMI)[N(CN)_2_] using the capillary action method [22]. As shown in Figure 8a–c, an increase in the filling level of (EMI)[N(CN)_2_] leads to a gradual decrease in the adsorbed nitrogen gas and the approach of the C–N rocking mode of the introduced EMI cation (Band **A** in Figure 8b) toward that of the bulk state (Band **B** in Figure 8b). The RT ionic conductivity at a volumetric filling factor *f* = 0.625 is as high as 4.4 × 10^−3^ S cm^−1^ (Figure 8d), which is the first IL@MOF hybrid to exhibit superionic conductivity (> 10^−3^ S cm^−1^). To the best of our knowledge, this value is still the highest RT ionic conductivity reported for IL@MOF hybrids to date. Additionally, (EMI)[N(CN)_2_] in the pores underwent no crystallization events (melting point of bulk (EMI)[N(CN)_2_]: –12°C [57]) down to the lowest measured temperature (ca. –120°C) due to the confinement effect, thereby showing the ionic conductivity higher than that of bulk (EMI)[N(CN)_2_] below RT (Figure 8e).

Whereas the enlarged mesopores of MOFs lead to an increase in ionic conductivity of ILs introduced into the MOF pores due to the increased fraction of the core region, the enlargement apparently impedes studies on the influence of ligand modifications on ionic conductivity.

### 5.2 Pore Surface State Dependence

In most IL@MOF hybrids, hydrogen‐bonding interactions probably occur between the component ions of the guest ILs and the framework of the host MOFs. Even in the TFSA anion, which has been most commonly used in studies of IL@MOF hybrids, the highly electronegative fluorine atoms form C–H··F hydrogen‐bonding host–guest interactions with a weak hydrogen‐bond donating CH moiety [58]. Host–guest interactions hinder ion diffusion, especially when the ILs occupy the vicinity of the pore surface.

**FIGURE 9:**
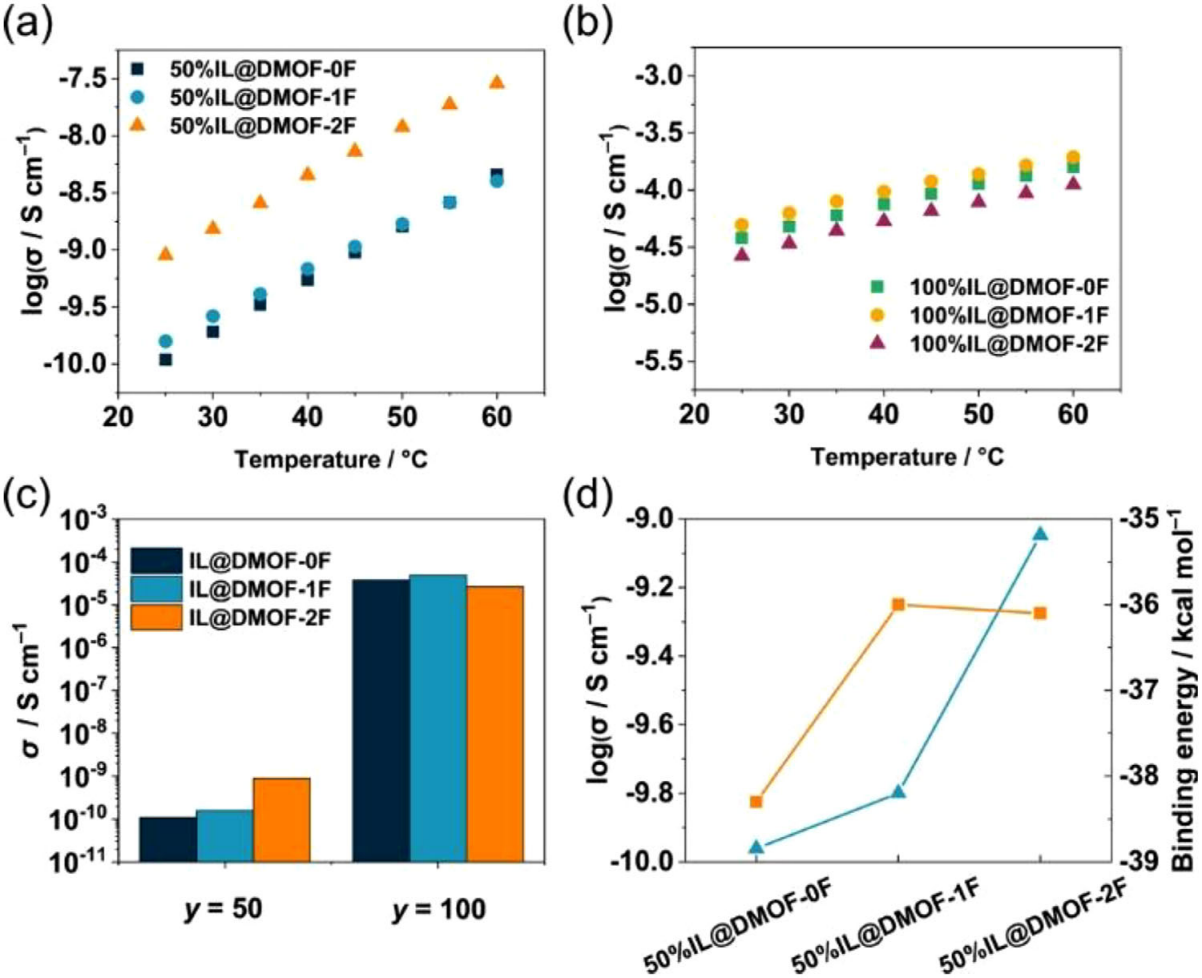
Temperature dependence of ionic conductivity (*σ*) of (EMI)(TFSA)@DMOF‐*x*Fs hybrids with (a) *f* = 0.5 and (b) *f* = 1.0. (c) Comparison of *σ* at 25°C for (EMI)(TFSA)@DMOF‐*x*Fs with *f* = 0.5 (*y* = 50) and *f* = 1.0 (*y* = 100). (d) Comparisons of *σ* at 25°C (pale blue triangles) and calculated binding energy (orange squares) for (EMI)(TFSA)@DMOF‐*x*Fs with *f* = 0.5. Adapted with permission from Ref. [60]. Copyright 2025 American Chemical Society.

Our group recently found that the ionic conductivity of (EMI)(TFSA) in pores varied depending on the number of hydrophobic fluorine atoms attached to the BDC ligand (*x*FBDC) in Zn‐based MOFs, [Zn_2_(*x*FBDC)(tmBDC)(DABCO)] (DMOF‐*x*F) [59], with one‐dimensional (1D) porous channels (pore size: ca. 0.6 nm) [60]. At a high filling level (*f* = 1.0), the RT ionic conductivity remained largely unchanged on the order of 10^−5^ S cm^−1^, regardless of the number of fluorine atoms. However, the RT ionic conductivity at a low filling level (*f* = 0.5) increases with increasing the number of fluorine atoms by approximately one order of magnitude (1.1 × 10^−10^ S cm^−1^ for *x* = 0 to 9.0 × 10^−10^ S cm^−1^ for *x* = 2) (Figure 9a–c). Given that a higher fluorine concentration on the pore surface leads to fewer hydrogen‐bonding interactions with ILs (especially the TFSA anion), ILs at a lower filling level preferentially occupy the core region to avoid obstructing ion migration in the pores. The decrease in host–guest interactions with increasing the number of fluorine atoms was confirmed by the binding energies calculated from Monte Carlo simulations (Figure 9d). While the primary objective of earlier studies on fluorinated MOFs has been to improve the selective CO_2_ gas adsorption and storage by utilizing the electrostatic F···C(CO_2_) interactions based on the quadrupole moment of CO_2_ [61, 62], this paper demonstrated that the fluorine substitution rationally controls the transport properties of ILs in the MOF pores.

On the other hand, the introduction of a hydrophilic amino group into the BDC ligand (i.e., BDC‐NH_2_) of UiO‐66(Zr) had little effect on the ionic conductivity of (BMI)(TFSA) in the pores [43].

## 6 Lithium‐Ion Conductivity

Because ILs are a highly ionic “electrolyte” with extremely low volatility (i.e., negligible flammability), lithium salts can be dissolved into them to obtain a lithium‐ion electrolyte (i.e., Li^+^‐containing IL) with a stable liquid state [63, 64]. Furthermore, the introduction of a Li^+^‐containing IL into MOF pores can readily produce a Li^+^‐ion‐conducting solid electrolyte, which is crucial for the development of all‐solid‐state lithium‐ion batteries [65] capable of preventing liquid leakage and vaporization (i.e., ignition and expansion). Although there are many issues to be solved for practical applications such as insufficient ionic conductivity and high production costs, the following introduces the current research status of the transport properties of Li^+^ ions in Li^+^‐containing IL@MOF hybrids, especially the Li^+^‐ion transport number (Table 1).

**TABLE 1:** Li^+^‐ion transport number ($t_{Li^{+}}$) of Li^+^‐containing ILs introduced into MOFs.

Li^+^‐containing IL	MOF	Dimensionality	Aperture size (nm)	$t_{Li^{+}}$	Refs.
Li_0.2_EMI_0.8_(TFSA)	UiO‐66(Zr)	3D	0.53	0.48	[27]
Li_0.34_EMI_0.66_(TFSA)	UiO‐66(Zr)	3D	0.53	ca. 0.25	[25]
Li_0.34_EMI_0.66_(TFSA)	UiO‐66(Zr)	3D	0.53	0.33	[26]
Li_0.2_EMI_0.8_(TFSA)	UiO‐66‐NH_2_(Zr)	3D	0.55	0.30	[29]
Li_0.17_EMI_0.83_(TFSA)	UiO‐67(Zr)	3D	0.83	0.13	[24]
Li_0.2_EMI_0.8_(TFSA)	UiO‐67(Zr)	3D	0.83	0.30	[27]
Li_0.2_EMI_0.8_(TFSA)	HKUST‐1(Cu)	3D	1.0, 1.4	0.46 (25°C) 0.62 (100°C)	[28]
Li_0.2_EMI_0.8_(TFSA)	MOF‐525(Cu)	3D	1.2 × 0.7	0.36	[23]
Li_0.23_DEME_0.77_(TFSA)	UiO‐66‐NH_2_(Zr)	3D	0.55	0.28	[29]

**FIGURE 10:**
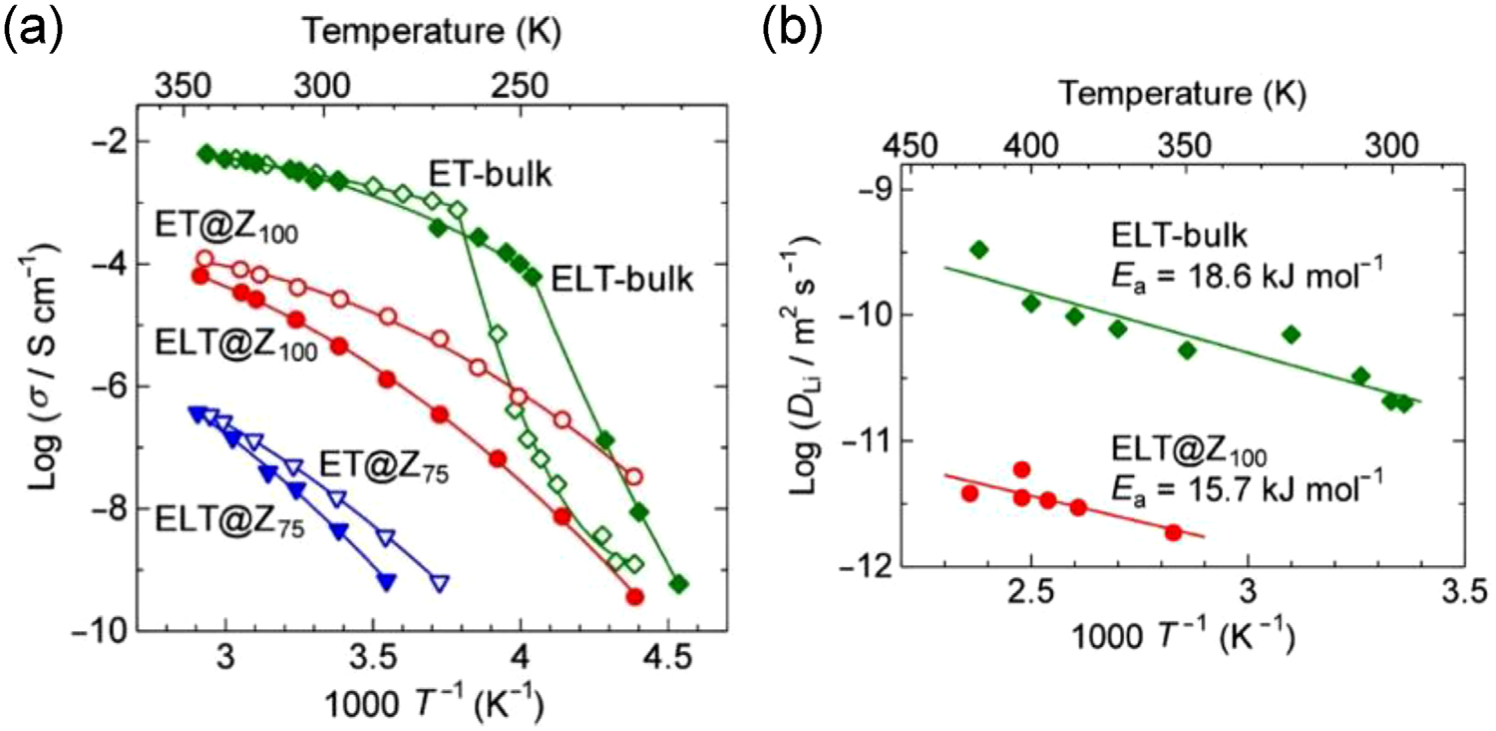
(a) Arrhenius plots of the ionic conductivity (*σ*) of (EMI)(TFSA)@ZIF‐8 with *f* = 0.75 (ET@Z_75_), (EMI)(TFSA)@ZIF‐8 with *f* = 1.0 (ET@Z_100_), Li_0.2_EMI_0.8_(TFSA)@ZIF‐8 with *f* = 0.75 (ELT@Z_75_), and Li_0.2_EMI_0.8_(TFSA)@ZIF‐8 with *f* = 1.0 (ELT@Z_100_), together with those of bulk (EMI)(TFSA) (ET‐bulk) and Li_0.2_EMI_0.8_(TFSA) (ELT‐bulk). The solid lines are drawn as guides for the eye. (b) Temperature dependence of the Li^+^‐ion diffusion coefficient (*D*_Li_) of ELT@Z_100_ and ELT‐bulk estimated from ^7^Li PFG‐NMR measurements, where the solid lines are fit to the Arrhenius equation. Adapted with permission from Ref. [68]. Copyright 2015 Royal Society of Chemistry.

Whereas Long et al. have reported that volatile organolithium compounds such as Li(O*i*Pr) [66] and Li(O*t*Bu) [67] introduced into microporous MOFs such as [Mg_2_(DOBDC)] (MOF‐74(Mg) or CPO‐27‐Mg) and UiO‐66(Zr) exhibited a moderately high ionic conductivity (*σ*_RT_ > 10^−4^ S cm^−1^), the first example of the introduction of Li^+^‐containing IL into MOF pores was reported by Fujie and Kitagawa et al. [68]. They dissolved Li(TFSA) in (EMI)(TFSA) in a molar ratio of Li^+^:EMI^+^ = 2:8, followed by the introduction of the Li^+^‐containing IL, Li_0.2_EMI_0.8_(TFSA), into the micropores of ZIF‐8 by dry milling. The vibrational modes assigned to the TFSA anion (e.g., the coupling mode of the S–N–S stretching and CF_3_ bending vibrations) suggest the formation of an ionic [Li(TFSA)_2_]^–^ species in the Li^+^‐containing IL [69, 70]. Given that the solvated ion is obviously larger than the aperture of ZIF‐8 (ca. 0.34 nm), it seems plausible that the RT ionic conductivity is approximately one order of magnitude lower than the (EMI)(TFSA)@ZIF‐8 hybrid free from Li^+^ ions (2.6 × 10^−5^ S cm^−1^ to 4.4 × 10^−6^ S cm^−1^) (Figure 10a). However, even in PCN‐777(Zr) with a wider aperture (ca. 2.6 nm), the ionic conductivity of the pore‐encapsulated Li*_x_* EMI_1–_ *_x_* [N(CN)_2_] gradually decreases with increasing Li^+^ content (*x*) (2.8 × 10^−3^ S cm^−1^ for *x* = 0 to 6.3 × 10^−5^ S cm^−1^ for *x* = 0.4) (Figure 7d) [53], indicating that the electrostatic interaction between Li^+^ ions and counter anions to form the neutralized ion pairs such as [Li{N(CN)_2_}_2_]^–^ is also considered to be an important factor reducing the ionic conduction.

Fujie and Kitagawa et al. estimated the diffusion coefficient of Li^+^ ions in the Li_0.2_EMI_0.8_(TFSA)@ZIF‐8 hybrid based on pulsed‐field gradient (PFG) NMR measurements (Figure 10b) [68]. Extrapolating the temperature dependence by the Arrhenius equation, the RT diffusion coefficient was determined to be approximately 10^−12^ m^2^ s^−1^, which is more than one order of magnitude lower than that of the bulk Li_0.2_EMI_0.8_(TFSA) (ca. 2 × 10^−11^ m^2^ s^−1^). Notably, the diffusivity of Li^+^ ions in bulk Li_0.2_EMI_0.8_(TFSA) was comparable to that of EMI cations (ca. 5 × 10^−11^ m^2^ s^−1^) and TFSA anions (ca. 3 × 10^−11^ m^2^ s^−1^) in bulk (EMI)(TFSA) [48].

The Li^+^‐ion transport number ($t_{Li^{+}}$) in the electrolytes can be readily estimated by the DC polarization method using a lithium symmetric cell. Whereas the $t_{Li^{+}}$ values of bulk Li^+^‐containing ILs, Li*_x_* EMI_1–_ *_x_* (TFSA) (*x* = 0.17–0.20), lie in the range of 0.11–0.18 [23, 24, 27, 29], the value shows a tendency to increase when Li*_x_* EMI_1–_ *_x_* (TFSA) is introduced into the MOF pores, as shown in Table 1. Many papers have stated that the smaller size of the Li^+^ ion (1.84 Å^3^) compared to the apertures connecting the pores, unlike the EMI cation (149 Å^3^) and TFSA anion (196 Å^3^), is a factor dominating the selective transportation of the Li^+^ ions in the pore space of MOFs. However, the aforementioned vibrational study confirmed the formation of an ionic [Li(TFSA)_2_]^–^ species in Li*_x_* EMI_1–_ *_x_* (TFSA), and therefore, it is likely that the Li^+^ ions pass through the apertures during hopping between TFSA anions in a manner similar to the Grotthuss mechanism [71, 72] in proton conduction [41, 73, 74]. As presented in Table 1, there is no straightforward correlation between the aperture size and $t_{Li^{+}}$ values. These $t_{Li^{+}}$ values determined from the DC polarization method are comparable to that predicted from MD simulations for the Li_0.2_BMI_0.8_(TFSA)@HKUST‐1 hybrid (ca. 0.3–0.4) [41] and typical values in electrolytes for lithium‐ion batteries (ca. 0.2–0.4). Although not a Li^+^‐containing IL, the $t_{Li^{+}}$ values higher than 0.6 have been observed in hybrids formed by introducing lithium salts (e.g., LiCl) into MOF pores because of the low diffusivity of the anions [75, 76, 77].

Currently, all Li^+^‐containing ILs for which the $t_{Li^{+}}$ values in the MOF pores have been reported utilize TFSA anions. However, for example, ab initio calculations predicted that the formation energy (from an isolated ion) of an ion pair, [Li(FSA)] (–552.7 kJ mol^−1^), is smaller than that of [Li(TFSA)] (–574.0 kJ mol^−1^) [78], although the FSA anion, which has recently attracted attention as an anion in electrolytes for lithium‐ion batteries [79], has a higher Lewis basicity than the TFSA anion. Thus, the acceleration of the Li^+^‐ion hopping event (i.e., increase in $t_{Li^{+}}$) by replacing the TFSA anion with the FSA anion is highly expected; in this context, high ionic conductivity (i.e., low internal resistance) in addition to electrochemical stability (i.e., wide potential window) is essential for applications in lithium‐ion batteries. Immobilization of anionic species in cationic MOFs [67] and reduction of electrostatic interactions by encapsulating Li^+^ ions with glycol ethers commonly called glymes ($t_{Li^{+}}$ > 0.2) [80, 81] are another promising approach to increase the $t_{Li^{+}}$ values.

To date, proton‐conducting MOF hybrids with inorganic acids, such as H_2_SO_4_ and H_3_PO_4_, introduced into the pores [82, 83], and Na^+^‐ion‐conducting MOF hybrids with Na^+^‐containing ILs introduced into the pores [47, 84, 85] have been reported, although they are not presented in this review.

## 7 Summary and Outlook

In this review, we present recent developments in the control of ionic conduction by designing and selecting the component species of ILs (cations and anions) and MOFs (metal ions and ligands) in IL‐introduced MOF hybrids. Although there are only a handful of studies to date in the literature demonstrating the RT ionic conductivity higher than 10^−3^ S cm^−1^, it seems that the ionic conduction of IL@MOF hybrids is merely a reflection of that of the bulk ILs in most cases. However, theoretical calculations have predicted that the transport number of the TFSA anion significantly decreases when a TFSA‐based IL is introduced into the MOF pores, and the influence of the pore/aperture size and pore surface potential of MOFs on the ionic conduction of ILs introduced in the pores remains largely unclear. In particular, a comprehensive exploration involving diverse combinations of ILs and MOFs will elucidate the ion‐conducting behavior of IL@MOF hybrids at low temperatures—the most characteristic behavior of IL@MOF hybrids—at which the corresponding bulk IL freezes. Given that the liquid properties of ILs can be controlled by judiciously designing and selecting the component ions (often called “designer solvents”), the combination of experimental and theoretical approaches is urgently required for rational exploration of new and more advanced ion‐conducting IL@MOF hybrids. For reference, the IL@MOF hybrids with reported ionic conductivity are summarized in Table 2 [20, 22, 39, 40, 42, 43, 55, 56, 86, 87, 88, 89, 90, 91].

**TABLE 2:** Ionic conductivity (*σ*) and activation energy (*E*_a_) of IL@MOFs.^a^

IL	MOF	Dimensionality	Introduction method^b^	Filling level	*σ* (S cm^−1^)	*E*_a_ (eV)	Refs.
(EMI)Br	MIL‐53‐NH_2_(Al)	1D	IS	*f* = 0.87	3.0 × 10^−5^ (80°C)	0.37	[86]
(EMI)(TFSA)	MIL‐121(Al)	1D‐like	CA	23 wt% (EMI)(TFSA)	ca. 5 × 10^−4^ (30°C)	0.25	[87]
(EMI)(TFSA)	Li‐MIL‐121(Al)	1D‐like	CA	23 wt% (EMI)(TFSA)	5 × 10^−4^ (30°C)	0.25	[87]
(EMI)(SCN)	MIL‐101(Cr)	3D	WI (MeOH)	*f* = 1.0	1.2 × 10^−3^ (25°C) 6.2 × 10^−3^ (150°C)	0.17	[55]
(EMI)[N(CN)_2_]	MIL‐101(Cr)	3D	WI (MeOH)	*f* = 1.0	4.1 × 10^−4^ (25°C) 2.5 × 10^−3^ (150°C)	0.18	[55]
(HSO_3_BMI)(HSO_4_)	MIL‐101(Cr)	3D	WI (EtOH)	*f* = 0.71	2.8 × 10^−6^ (50°C)	0.21	[39]
(HSO_3_BMI)(HSO_4_)	MIL‐101(Cr)	3D	SIB (H_2_O)	*f* = 0.95	4.4 × 10^−2^ (50°C)	0.09	[39]
(DMI)(BF_4_)	MIL‐101(Cr)	3D	WI (MeOH)	*f* = 1.0	1.3 × 10^−4^ (20°C) 3.7 × 10^−3^ (100°C)	0.40	[88]
(AMI)Cl	MIL‐101(Cr)	3D	WI (MeOH)	*f* = 1.0	1.8 × 10^−4^ (20°C) 2.7 × 10^−3^ (100°C)	0.32	[88]
(EMI)Cl	MIL‐101‐SO_3_H(Cr)	3D	WI (H_2_O)	42 wt% (EMI)Cl	1.8 × 10^−3^ (25°C) 1.4 × 10^−1^ (70°C)	0.22	[89]
(BMI)(TFSA)	HKUST‐1	3D	WI (MeCN)	*f* = 0.15	ca. 4.5 × 10^−4^ S cm^2^ mol^−1^ (RT)		[40]
(BMI)(TFSA)	HKUST‐1	3D	CA	*f* = 0.80	ca. 8 × 10^−7^ (RT)		[42]
(EMI)(TFSA)	ZIF‐8	3D	CA	*f* = 1.0	2.6 × 10^−5^ (22°C) 1.2 × 10^−4^ (68°C)	0.35	[20]
(EMI)(TFSA)	DMOF‐0F	1D	CA	*f* = 1.0	3.8 × 10^−5^ (25°C)	0.38	[60]
(EMI)(TFSA)	DMOF‐1F	1D	CA	*f* = 1.0	5.0 × 10^−5^ (25°C)	0.36	[60]
(EMI)(TFSA)	DMOF‐2F	1D	CA	*f* = 1.0	2.7 × 10^−5^ (25°C)	0.37	[60]
(BMI)(TFSA)	UiO‐66(Zr)	3D	WI (MeCN)	*f* = 0.15	ca. 4 × 10^−8^ (RT)		[43]
(Et_4_N)(TFSA)^c^	UiO‐66(Zr)	3D	CA	33.3 wt% (Et_4_N)(TFSA)	2.2 × 10^−8^ (22°C) 5.9 × 10^−5^ (90°C)	0.96	[56]
(BMI)(TFSA)	UiO‐66‐NH_2_(Zr)	3D	WI (MeCN)	*f* = 0.15	ca. 3 × 10^−8^ (RT)		[43]
(BMI)(TFSA)	DUT‐52(Zr)	3D	WI (MeCN)	*f* = 0.15	ca. 7 × 10^−8^ (RT)		[43]
(BMI)(TFSA)	UiO‐67(Zr)	3D	WI (MeCN)	*f* = 0.15	ca. 9 × 10^−8^ (RT)		[43]
(Et_4_N)(TFSA)^c^	UiO‐67(Zr)	3D	CA	42.8 wt% (Et_4_N)(TFSA)	5.5 × 10^−8^ (22°C) 6.1 × 10^−5^ (90°C)	0.92	[56]
(EMI)Cl	UiO‐67(Zr)	3D	CA	[(EMI)Cl]/[UiO‐67(Zr)] = 7.82	1.5 × 10^−4^ (100°C) 1.7 × 10^−3^ (200°C)	0.37	[90]
(HSO_3_PEI)(MeSO_3_) + MeSO_3_H	UiO‐67(Zr)	3D	CA	*f* = 1.0	2.2 × 10^−5^ (20°C)	0.32	[91]
(Et_4_N)(TFSA)^c^	PCN‐56(Zr)	3D	CA	46.6 wt% (Et_4_N)(TFSA)	9.4 × 10^−8^ (22°C) 7.7 × 10^−5^ (90°C)	0.88	[56]
(BMI)(TFSA)	UiO‐68‐NH_2_(Zr)	3D	WI (MeCN)	*f* = 0.15	ca. 1 × 10^−7^ (RT)		[43]
(EMI)[N(CN)_2_]	PCN‐777(Zr)	3D	CA	*f* = 0.625	4.4 × 10^−3^ (25°C) 1.5 × 10^−2^ (130°C)	0.20	[22]

^a^ Hybrids containing H^+^, Li^+^, and Na^+^ ions in ILs and neutral guest molecules were excluded.

^b^ IS, ionothermal method; WI, wet impregnation method; CA, capillary action method; SIB, ship‐in‐bottle method.

^c^ Note that the melting point of (Et_4_N)(TFSA) (102°C) exceeds 100°C, which is often used to define IL.

## Conflicts of Interest

The authors declare no conflicts of interest.

## Data Availability

The data that support the findings of this study are available from the corresponding author upon reasonable request.
